# Melatonin inhibits ESCC tumor growth by mitigating the HDAC7/β-catenin/c-Myc positive feedback loop and suppressing the USP10-maintained HDAC7 protein stability

**DOI:** 10.1186/s40779-022-00412-0

**Published:** 2022-09-27

**Authors:** Zhi-Qiang Ma, Ying-Tong Feng, Kai Guo, Dong Liu, Chang-Jian Shao, Ming-Hong Pan, Yi-Meng Zhang, Yu-Xi Zhang, Di Lu, Di Huang, Fan Zhang, Jin-Liang Wang, Bo Yang, Jing Han, Xiao-Long Yan, Yi Hu

**Affiliations:** 1 Department of Medical Oncology, Senior Department of Oncology, the Fifth Medical Center, Chinese PLA General Hospital, Beijing, 100853 China; 2 Department of Thoracic Surgery, Tangdu Hospital, the Fourth Military Medical University, Xi’an, 710038 China; 3 Department of Cardiothoracic Surgery, the 71th Group Army Hospital of PLA, the Affiliated Huaihai Hospital of Xuzhou Medical University, Xuzhou, 221004 Jiangsu China; 4 Department of Thoracic Surgery, Shaanxi Provincial People’s Hospital, the Third Affiliated Hospital of Xi’an Jiaotong University, Xi’an, 710068 China; 5 State Key Laboratory of Cardiovascular Disease, Fuwai Hospital, National Center for Cardiovascular Diseases, Chinese Academy of Medical Sciences, Peking Union Medical College, Beijing, 100037 China; 6 Department of Ophthalmology, Tangdu Hospital, the Fourth Military Medical University, Xi’an, 710038 China; 7 Department of Cardiovascular Surgery, Xijing Hospital, the Fourth Military Medical University, Xi’an, 710032 China

**Keywords:** Melatonin, Histone deacetylase 7 (HDAC7), β-catenin, c-Myc, Ubiquitin-specific peptidase 10 (USP10), Esophageal squamous cell carcinoma (ESCC)

## Abstract

**Background:**

Melatonin, a natural hormone secreted by the pineal gland, has been reported to exhibit antitumor properties through diverse mechanisms of action. However, the oncostatic function of melatonin on esophageal squamous cell carcinoma (ESCC) remains elusive. This study was conducted to investigate the potential effect and underlying molecular mechanism of melatonin as single anticancer agent against ESCC cells.

**Methods:**

ESCC cell lines treated with or without melatonin were used in this study. In vitro colony formation and EdU incorporation assays, and nude mice tumor xenograft model were used to confirm the proliferative capacities of ESCC cells. RNA-seq, qPCR, Western blotting, recombinant lentivirus-mediated target gene overexpression or knockdown, plasmids transfection and co-IP were applied to investigate the underlying molecular mechanism by which melatonin inhibited ESCC cell growth. IHC staining on ESCC tissue microarray and further survival analyses were performed to explore the relationship between target genes’ expression and prognosis of ESCC.

**Results:**

Melatonin treatment dose-dependently inhibited the proliferative ability and the expression of histone deacetylase 7 (HDAC7), c-Myc and ubiquitin-specific peptidase 10 (USP10) in ESCC cells (*P* < 0.05). The expressions of HDAC7, c-Myc and USP10 in tumors were detected significantly higher than the paired normal tissues from 148 ESCC patients (*P* < 0.001). Then, the Kaplan–Meier survival analyses suggested that ESCC patients with high HDAC7, c-Myc or USP10 levels predicted worse overall survival (Log-rank *P* < 0.001). Co-IP and Western blotting analyses further revealed that HDAC7 physically deacetylated and activated β-catenin thus promoting downstream target *c-Myc* gene transcription. Notably, our mechanistic study validated that HDAC7/β-catenin/c-Myc could form the positive feedback loop to enhance ESCC cell growth, and USP10 could deubiquitinate and stabilize HDAC7 protein in the ESCC cells. Additionally, we verified that inhibition of the HDAC7/β-catenin/c-Myc axis and USP10/HDAC7 pathway mediated the anti-proliferative action of melatonin on ESCC cells.

**Conclusions:**

Our findings elucidate that melatonin mitigates the HDAC7/β-catenin/c-Myc positive feedback loop and inhibits the USP10-maintained HDAC7 protein stability thus suppressing ESCC cell growth, and provides the reference for identifying biomarkers and therapeutic targets for ESCC.

**Supplementary Information:**

The online version contains supplementary material available at 10.1186/s40779-022-00412-0.

## Background

Melatonin, a hormone mainly secreted from the pineal gland, has been proven to possess extensive biological activities including circadian rhythms regulation, anti-oxidation, anti-inflammation and immunoregulation [1–5]. As to cancer, mounting evidence indicates that melatonin exhibits oncostatic features at the initiation, progression and metastasis phases through multiple and interrelated mechanisms [5–11]. Esophageal squamous cell carcinoma (ESCC) is ranked as the 6th leading cause of cancer-related deaths in the world [12, 13]. Like many other solid tumors, the ESCC development and progression are considered as a multiple-step process caused by the accumulation of dysregulated oncogenes [13]. Though melatonin has emerged as an appealing anti-cancer agent against various malignancies, its role and underlying molecular mechanism on ESCC remain elusive.

Histone deacetylase 7 (HDAC7), a member of the HDAC IIa subfamily (HDAC4, 5, 7 and 9), deacetylates both histones and non-histone proteins and plays important roles in cancer progression [14]. High level of HDAC7 is frequently associated with advanced cancers and contributes to the poor prognosis of lung and breast cancer patients [15, 16]. Nevertheless, the clinical significance of HDAC7 on ESCC have not yet been evaluated. Previous studies revealed that ectopic HDAC7 expression enhanced cancer cell proliferation, angiogenesis and metastasis [15–19]. Mechanistically, HDAC7 directly binds and deacetylates signal transducer and activator of transcription 3 (STAT3) and β-catenin thus regulating the expression of downstream oncogenes [15, 17]. In the canonical Wnt/β-catenin pathway, β-catenin is dephosphorylated and activated after Wnt signaling activation [17, 20]. Interestingly, recent studies confirmed that β-catenin deacetylation also led to β-catenin nuclear import thus directly enhancing downstream *c-Myc* oncogene transcription [17, 21]. Therefore, whether the potential HDAC7/β-catenin/c-Myc axis involves in regulation of the ESCC growth warrants further investigation.

Ubiquitin-specific peptidase 10 (USP10) is a highly conserved deubiquitinating enzyme, and is extensively involved in the progression of a broad spectrum of cancers [22, 23]. Previous studies demonstrated that USP10 could deubiquitinate and stabilize Yes-associated protein (YAP)/PDZ-binding motif (TAZ), HDAC6, FMS-like tyrosine kinase 3 (FLT3), spleen tyrosine kinase (SYK) and topoisomerase Iiα (TOP2α), thus promoting cancer progression [22–26]. Since HDAC7 shares similar structures with HDAC6, HDAC7 may be a new potential substrate of USP10. Interestingly, Cai et al. [27] demonstrated that melatonin could decrease USP10 expression thus antagonizing premature senescence of cardiac progenitor cells. Moreover, melatonin reportedly worked as a potential HDAC IIa inhibitor and mitigated lung cancer cell proliferation and metastasis [1]. Hence, we hypothesize that melatonin may inhibit the USP10 and HDAC7 signaling thus performing the oncostatic actions. Here in this study, we explored the expression and prognostic role of HDAC7/USP10/c-Myc on ESCC patients, and further investigated the potential action and molecular mechanism of melatonin as single anticancer agent in ESCC cell models.

## Methods

### Cell culture and cell treatment

Human ESCC cell lines (EC109, EC9706, KYSE150 and TE-1) and HEK-293T were obtained from the Hunan Fenghui Biotechnology Co., Ltd. (Changsha, China). These cells were cultured in high glucose DMEM medium (C11995500, Gibco, NY, USA) supplemented with 10% fetal bovine serum (04-001-1A, FBS, Biological Industries, Israel) and penicillin–streptomycin solution (100 U/ml, P1400, Solarbio, Beijing, China). Melatonin (#14427, Cayman Chemical Company, Michigan, USA), SAHA (HY-10221, MedChemExpress, USA), Lf3 (HY-101486, MedChemExpress, USA) and 10058-F4 (HY-12702, MedChemExpress, USA) stock solution was prepared in DMSO and diluted in culture media immediately prior to the subsequent experiments. The ESCC cells were treated with these chemical compounds for 48 h and then harvested for further analyses.

### Colony formation assay

Eight hundred cells were seeded in the 6-well plate. After being given the assigned treatments for 48 h, the cells were further cultured for about 12 d. Then, colonies were fixed with formalin and stained with 0.1% crystal violet (C8470, Solarbio, Beijing, China). The colonies containing more than 50 cells were counted for further analyses.

### 5-Ethynyl-2′-deoxyuridine (EdU) incorporation assay

BeyoClick™ EdU-555 fluorescence staining detection kit (Beyotime, Shanghai, China) was used for detecting the newly synthesized DNA and evaluating cell proliferation according to the manufacturer’s instructions. Images were obtained by the FV1000 confocal microscope (Olympus, Japan).

### RNA-seq analysis

Total RNA was extracted from the control and melatonin (1 mmol/L, 2 mmol/L, 4 mmol/L) treatment groups of EC109 cells using TRIzol reagent (Invitrogen). Later, all the samples were sent to Gene Denovo Corporation (Guangzhou, China) for further RNA-seq detection and analyses via Illumina HiSeqTM 2500 (San Francisco, USA). The data were analyzed in depth by the Omicshare network platform of Gene Denovo (https://www.omicshare.com/).

### Western blotting

The standard Western blotting procedures were presented as previously described [28]. The listed primary antibodies were diluted by 1:1000 in this study, and the detailed information of these antibodies was listed in the Additional file 1: Table S1. Moreover, the 1:5000 diluted HRP-linked anti-IgG was used as the secondary antibody (Zhongshan Company, Beijing, China).

### Real-time quantitative polymerase chain reaction (qPCR)

The detailed procedure was conducted according to that previously described [28]. The primer sequences involved in qPCR are as follows: HDAC7, forward GAAAGAACAGTCCATCCCAACA, reverse GCTTATAGCGCAGCTTCAGG; GAPDH, forward TGACTTCAACAGCGACACCCA, reverse CACCCTGTTGCTGTAGCCAAA.

### ESCC patient samples and tissue microarray

One hundred and forty-eight ESCC patients who underwent esophagus surgery at the Tangdu Hospital between January 2012 and August 2015 were included in this study with the approval of the ethics committee of the Tangdu Hospital (TDLL-202110-02). Key inclusion criteria are ESCC patients with pT1-4N0-3M0 [the American Joint Committee on Cancer (AJCC) 7th edition], 18–80 years of age, performance status ≤ 1, no therapy for esophageal cancer before surgery and esophageal carcinoma radical surgery (R0) by open or minimally invasive surgery, while key exclusion criteria are those died from postoperative complications, with known active multiple cancers, psychosis or psychiatric symptoms inappropriate for participating the trial. The 148 pairs of formalin-fixed ESCC tumor tissues and corresponding adjacent normal tissues were made into paraffin-embedded tissue microarray. The complete follow-up was updated until death or August 2020, whichever came first.

### Immunohistochemistry (IHC) staining

IHC staining was performed on tissue microarray sections using the anti-HDAC7 (1:50, #33418, CST), anti-c-Myc (1:100, GB13076, Servicebio) and anti-USP10 (1:100, bs-9267R, Bioss) primary antibodies, and the standard protocols were followed as previously described [28]. Every tissue was equally divided into 4 visual fields in order to get its final average score. The IHC staining scores were classified based on the following criteria: 1) the percentage of positive cells was classified into four degrees (0, ≤ 5%; 1, 6–25%; 2, 26–50%; 3, 51–75%; and 4, > 75%); 2) the staining intensity was classified into four degrees (0, negative; 1, weak; 2, moderate; and 3, strong); then the scores were multiplied to get the final score, and case diagrams were listed in the Additional file 1: Fig. S1. The mean was used to classify the levels of HDAC7, USP10 or c-Myc expression.

### Lentivirus infection

The lentiviruses overexpressing or knocking down HDAC7, c-Myc and USP10 expression, and relevant empty vector lentiviruses were obtained from Genechem (Shanghai, China). The infection of lentivirus on the EC109 and EC9706 cells was conducted according to the protocol of the Genechem Recombinant Lentivirus Operation Manual provided by Genechem Corporation.

### In vivo tumor xenograft assays

The animal experiments were conducted on healthy adult male BALB/c athymic nude mice (6–8 weeks) obtained from the Laboratory Animal Center of the Fourth Military Medical University. Ten mice were randomly assigned to two experiments (*n* = 5 for each experiment): experiment (1) NC group and HDAC7 OE group; experiment (2) shCon and shUSP10 group. Different groups of the 7 × 10^6^ EC109 cells were separately subcutaneously inoculated into the left/right lower back of the nude mice (*n* = 5) for in vivo xenograft assay. After 21 to 27 d, the mice were all sacrificed at the same time, and the tumors were excised and weighted for further analysis. Housing and all other procedures were performed according to protocols approved by the Animal Experimentation Ethics Committee of the aforementioned university (IACUC-20200521). All animals received humanitarian care and study protocols were complied with the institution’s guidelines.

### Plasmid transfection

Plasmid transfection was performed using PEI (Polysciences) and Opti-MEM medium (Gibco, NY, USA). Plasmids pcDNA3-Flag-HDAC7, pcDNA3-HA-HDAC7, pcDNA3-Flag-β-catenin, pcDNA3-Flag-USP10, His-ubiquitin were purchased from SinoBiological (China). Plasmids pcDNA3-Flag-USP2, pcDNA3-Flag-USP4, pcDNA3-Flag-USP5, pcDNA3-Flag-USP7, pcDNA3-Flag-USP13, pcDNA3-Flag-USP14, pcDNA3-Flag-USP22, and flag-tagged mutant USP10 C424A, GST-tagged full-length or deletion mutants USP10 1–99, 100–399, 400–700, 400–798 were all kindly supported by Dr. Yizeng Fan in the First Affiliated Hospital of Xi’an Jiaotong University, Xi’an, China.

### Immunoprecipitation (IP)

The normal immunoglobin G (IgG, Millipore) was applied as the negative control. For the exogenous IP, per 1 mg whole cell lysates were incubated with 8 μg anti-Flag agarose gel (A-2220, sigma) at 4 °C for 4 h. For the endogenous IP, the lysates were incubated with 8 μg indicated immunoprecipitation antibody overnight at 4 °C, then further incubated with 40 μl PuroProteome Protein G Magnetic Beads (LSKMAGG02, Millipore) for 2 h. Later, the immunocomplexes were washed 4 times with NETN buffer, then diluted with the loading buffer and boiled for further immunoblot.

### Fractionation of cytoplasmic and nuclear extracts

The nuclear-cytosolic protein isolation kit (P0027, Beyotime, Shanghai, China) was applied to obtain the cytosolic and nuclear protein extraction according to the manufacturer’s instruction. Later, the fractions were respectively diluted with the loading buffer and boiled for further immunoblot.

### Statistical analyses

SPSS 23.0 (SPSS Inc., Chicago, USA) software was used to analyze the data. The χ^2^ test or Fisher’s exact test was used to assess the relationships between HDAC7/USP10/c-Myc expression and clinicopathological parameters of the ESCC patients. Kaplan–Meier plots were used for overall survival rates, then compared with the log-rank test. Univariate or multivariate survival analysis was carried out using the Cox proportional hazards model. Spearman correlation analyses were performed to analyze the relation between HDAC7/USP10/c-Myc expression in ESCC tumor tissues. Comparisons between two groups were performed by Student's *t*-test. Data are presented as the mean ± SEM or *n* (%). Statistical significance was set at *P* < 0.05.

## Results

### Melatonin treatment inhibits cell proliferation, and downregulates HDAC7 and c-Myc expression in ESCC cells

**Fig. 1:**
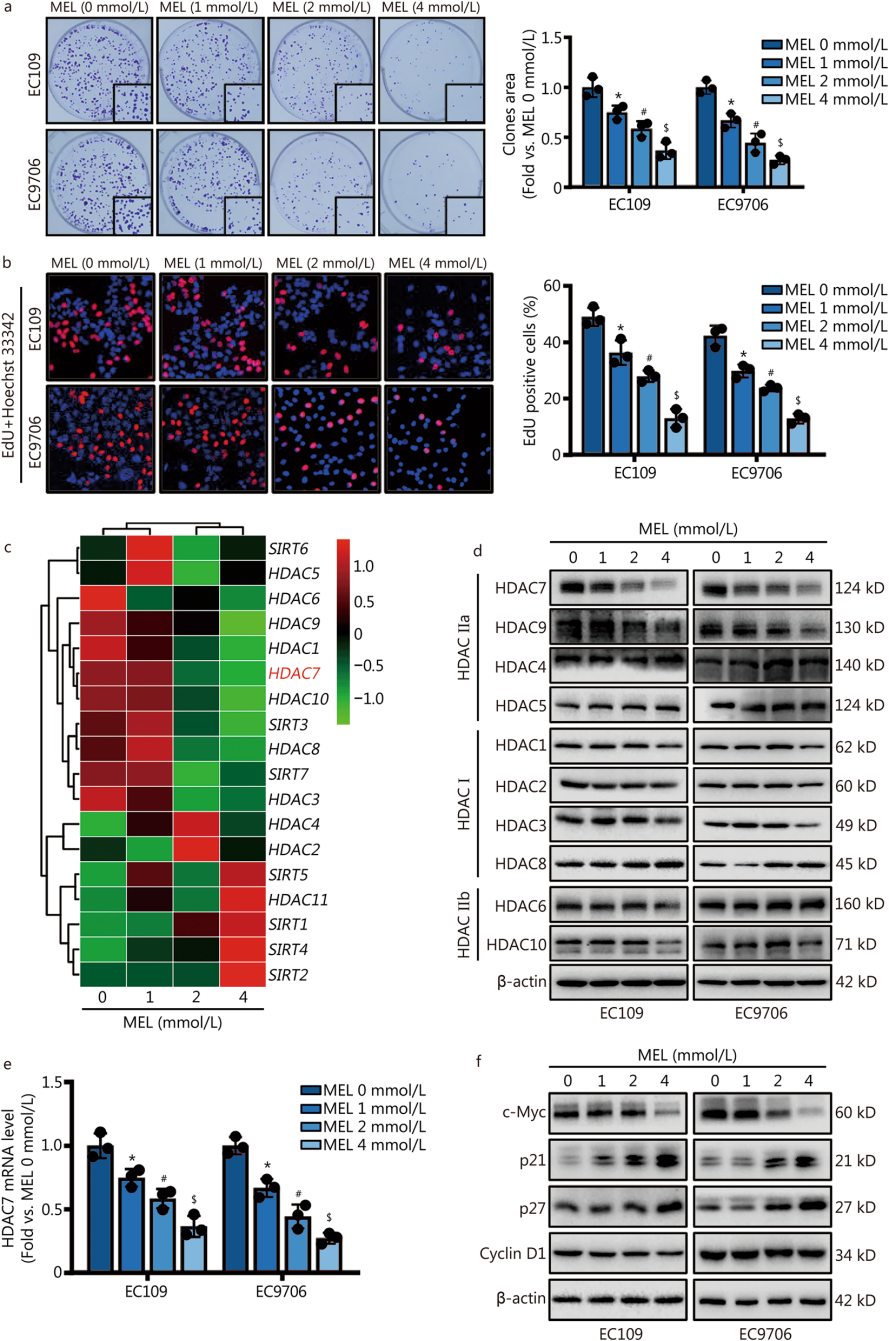
Melatonin induces cell proliferation inhibition, HDAC7 and c-Myc downregulation on ESCC cells. **a** Colony formation assay with melatonin pretreatments for 48 h. **b** Representative images and results of EdU incorporation assay. The result was calculated as the ratio between the number of EdU-stained cells (red fluorescence) and the total number of Hoechst 33342-stained cells (blue fluorescence). **c** Representative heatmap of HDACs gene expression based on the RNA‐seq results of EC109 cells. **d** Representative Western blotting results of HDACs. **e** Representative result of HDAC7 mRNA level. **f** Representative Western blotting results of c-Myc, p21, p27 and Cyclin D1. ^*^P < 0.05 vs. the MEL 0 mmol/L group, ^#^*P* < 0.05 vs. the MEL 1 mmol/L group, ^$^*P* < 0.05 vs. the MEL 2 mmol/L group. HDAC7 histone deacetylase 7, ESCC esophageal squamous cell carcinoma, MEL melatonin

To explore the action of melatonin treatment on the growth of ESCC cells, we conducted the colony formation and EdU incorporation assays to analyze the proliferative activities of EC109 and EC9706 cells. Melatonin (0 mmol/L, 1 mmol/L, 2 mmol/L or 4 mmol/L) treatment for 48 h significantly inhibited ESCC EC109 and EC9706 cells growth in a dose-dependent manner (*P* < 0.05, Fig. 1a, b), indicating melatonin is a potential oncostatic agent against ESCC. To further investigate the underlying molecular mechanisms through which melatonin exerted anti-proliferative effects on ESCC, the RNA-seq-based transcriptome analysis was used to assess the transcriptome changes among control and melatonin treatment (1 mmol/L, 2 mmol/L or 4 mmol/L) groups of EC109 cells. After analyzing the significant gene changes, we focused on the HDAC family and listed all the 18 genes in the heatmap (Fig. 1c). Aberrant HDACs expression contributes to the cancer development and progression. Interestingly, we found *HDAC7* was one of the top-ranked differentially expressed genes among the RNA-seq data (Fig. 1c). Moreover, both qPCR and Western blotting analyses double validated that melatonin significantly downregulated the HDAC7 mRNA and protein levels without influencing other HDACs’ expression in the ESCC cells (*P* < 0.05, Fig. 1d, e). In addition, the expression of genes which were involved in the regulation of cell cycle and proliferation were further tested after melatonin treatment. The expression of p21 and p27 is negatively regulated by c-Myc, and activation of c-Myc could directly prohibit the transcription of p21 and p27 [18, 29, 30]. Western blotting analyses showed the dose-dependent reduction of c-Myc but increase of p21 and p27 protein after melatonin treatment on the ESCC cells (Fig. 1f). However, melatonin did not affect the Cyclin-dependent kinase (CDK) 1/2/4/6 and the Cyclin A2/B1/D1/E1 protein levels in the ESCC cells (Fig. 1f, Additional file 1: Fig. S2). Taken together, these results suggest that melatonin can decrease ESCC cell proliferation, and is a potential HDAC7 and c-Myc pathway inhibitor.

### High HDAC7 expression predicts poor ESCC prognosis and promotes ESCC cell proliferation

**Fig. 2:**
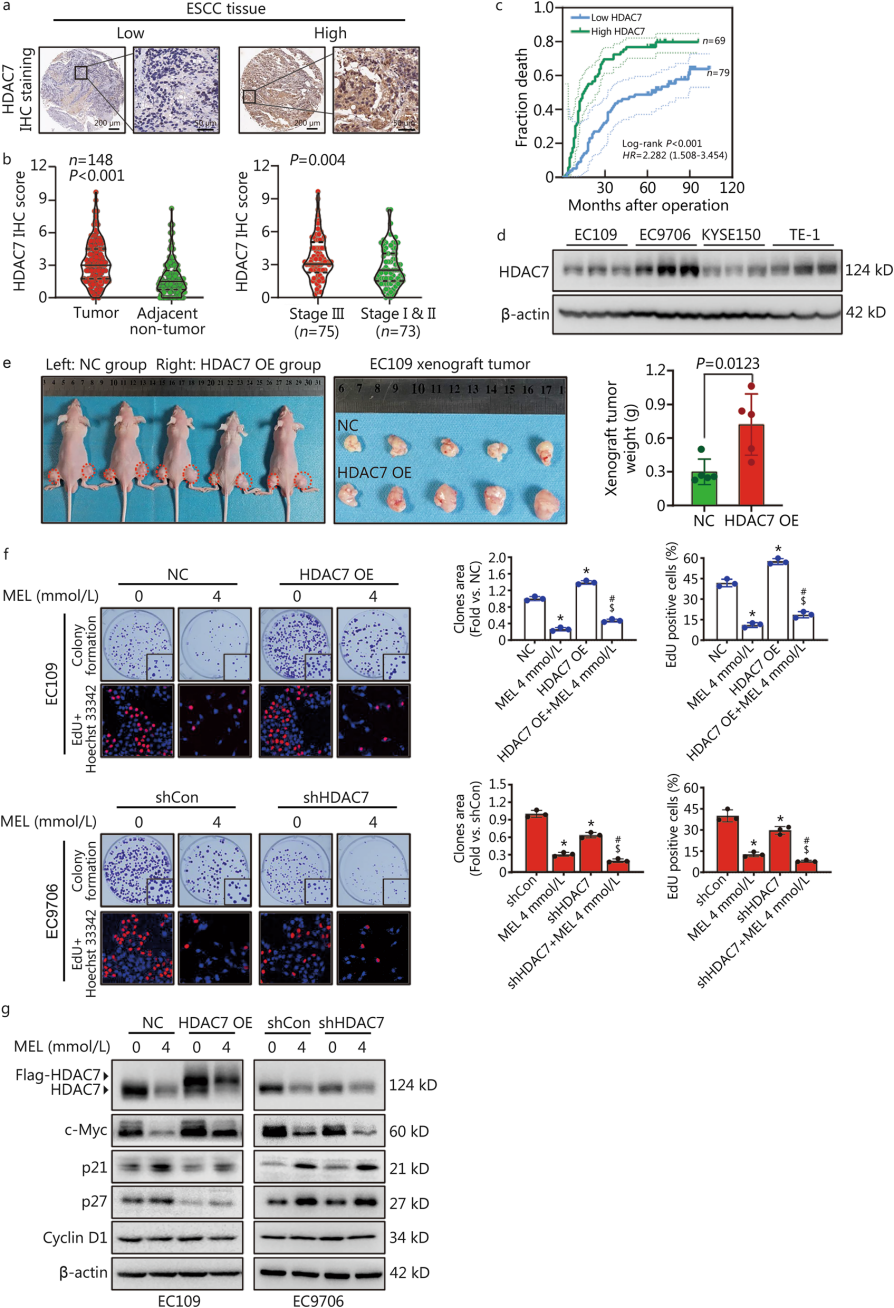
High HDAC7 expression predicts poor prognosis of ESCC patients and melatonin inhibits ESCC proliferation via inhibiting HDAC7. **a** Representative IHC images for HDAC7 expression in ESCC tissues. Scale bar, 200 μm and 50 μm (inset), respectively. **b** Statistical analysis of HDAC7 expression in 148 ESCC patients through IHC staining. **c** Kaplan–Meier survival analysis by the high/low HDAC7 levels of the 148 ESCC patients based on the microarray tissue IHC results. **d** Representative HDAC7 protein level in different ESCC cell lines. **e** Photographs and results showing xenograft tumor morphologies and weights in each group after subcutaneously injection of NC or HDAC7 OE EC109 cells, respectively. **f** Representative images and results of colony formation and EdU incorporation assays. **g** Representative Western blotting results of HDAC7, c-Myc, p21, p27 and Cyclin D1. ^*^P < 0.05 vs. the NC group or shCon group, ^#^*P* < 0.05 vs. the MEL 4 mmol/L group, ^$^*P* < 0.05 vs. the HDAC7 OE or shHDAC7 group. HDAC7 histone deacetylase 7, ESCC esophageal squamous cell carcinoma, MEL melatonin, OE overexpression, IHC immunohistochemistry

The prognostic role of HDAC7 expression in ESCC has not been reported. To confirm the HDAC7 expression in ESCC, the HDAC7 protein level was detected by IHC analysis in ESCC tissue microarray containing 148 paired tumor-normal tissues (Fig. 2a). We found the HDAC7 level was positively correlated to pTNM stage instead of differentiation, tumor invasion and lymphatic metastasis (Table 1). Moreover, the HDAC7 level in ESCC was significantly higher than that of normal tissues (*P* < 0.001, Fig. 2b), and ESCC tissues with high pTNM stage (III) had higher HDAC7 expression than those with low pTNM stage (I & II) (*P* = 0.004, Fig. 2b). Furthermore, the Kaplan–Meier analysis result showed that ESCC patients with high HDAC7 level were associated with worse overall survival (Log-rank *P* < 0.001, Fig. 2c). In addition, both univariate and multivariate Cox survival analyses suggested that HDAC7 was an independent prognostic factor for ESCC patients (*HR* = 2.200, 95% CI 1.471–3.290, *P* < 0.001; *HR* = 1.999, 95% CI 1.321–3.027, *P* = 0.001, Table 2).

**Table 1:** Association of HDAC7/USP10/c-Myc expression with clinicopathological parameters of patients with ESCC [*n *(%)]

Category	Total (*n* = 148)	HDAC7 expression			USP10 expression			c-Myc expression		
		Low (*n* = 79)	High (*n* = 69)	*P-*value	Low (*n* = 72)	High (*n* = 76)	*P-*value	Low (*n* = 73)	High (*n* = 75)	*P-*value
Age				0.154			0.301			0.669
< 60 years	45 (30.4)	28 (35.4)	17 (24.6)		19 (26.4)	26 (34.2)		21 (28.8)	24 (32.0)	
≥ 60 years	103 (69.6)	51 (64.6)	52 (75.4)		53 (73.6)	50 (65.8)		52 (71.2)	51 (68.0)	
Gender				0.982			0.156			0.734
Male	120 (81.1)	64 (81.0)	56 (81.2)		55 (76.4)	65 (85.5)		60 (82.2)	60 (80.0)	
Female	28 (18.9)	15 (19.0)	13 (18.8)		17 (23.6)	11 (14.5)		13 (17.8)	15 (20.0)	
Tumor location				0.388			0.381			0.294
Upper	8 (5.4)	6 (7.6)	2 (2.9)		4 (5.6)	4 (5.3)		6 (8.2)	2 (2.6)	
Middle	76 (51.4)	38 (48.1)	38 (55.1)		41 (56.9)	35 (46.0)		35 (48.0)	41 (54.7)	
Lower	64 (43.2)	35 (44.3)	29 (42.0)		27 (37.5)	37 (48.7)		32 (43.8)	32 (42.7)	
Tumor invasion				0.075			0.177			0.811
T1–T2	13 (8.8)	10 (12.7)	3 (4.3)		4 (5.6)	9 (11.8)		6 (8.2)	7 (9.3)	
T3–T4	135 (91.2)	69 (87.3)	66 (95.7)		68 (94.4)	67 (88.2)		67 (91.8)	68 (90.7)	
Lymphatic metastasis				0.080			0.001			0.002
N0	80 (54.1)	48 (60.8)	32 (46.4)		49 (68.1)	31 (40.8)		49 (67.1)	31 (41.3)	
N1–N3	68 (45.9)	31 (39.2)	37 (53.6)		23 (31.9)	45 (59.2)		24 (32.9)	44 (58.7)	
Differentiation				0.944			0.905			0.500
Well and moderate	129 (87.2)	69 (87.3)	60 (87.0)		63 (87.5)	66 (86.8)		65 (89.0)	64 (85.3)	
Poor	19 (12.8)	10 (12.7)	9 (13.0)		9 (12.5)	10 (13.2)		8 (11.0)	11 (14.7)	
pTNM stage				0.047			0.001			0.003
I–II	73 (49.3)	45 (57.0)	28 (40.6)		46 (63.9)	27 (35.5)		45 (61.6)	28 (37.3)	
III	75 (50.7)	34 (43.0)	41 (59.4)		26 (36.1)	49 (64.5)		28 (38.4)	47 (62.7)

**Table 2:** Univariate and multivariate analyses of prognostic factors for ESCC patients’ survival

Variables	Univariate analysis			Multivariate analysis		
	*HR*	95% CI	*P-*value	*HR*	95% CI	*P-*value
Age (≥ 60 years/ < 60 years)	1.264	0.816–1.959	0.294			
Gender (Male/female)	1.068	0.641–1.781	0.800			
Differentiation (Poor/well and moderate)	1.061	0.580–1.943	0.847			
Lymphatic metastasis (N1 – N3/N0)	1.855	1.265–2.808	0.002	1.386	0.902–2.132	0.137
pTNM stage (III/I – II)	1.812	1.213–2.707	0.004	1.541	1.126–2.110	0.007
HDAC7 expression (High/low)	2.200	1.471–3.290	< 0.001	1.999	1.321–3.027	0.001
USP10 expression (High/low)	2.799	1.843–4.250	< 0.001	1.725	1.032–2.885	0.038
c-Myc expression (High/low)	1.255	1.147–1.373	< 0.001	2.233	1.376–3.625	0.001

To further verify the function of HDAC7 on regulating ESCC proliferation, we checked the basal HDAC7 expression level in different ESCC cell lines (Fig. 2d), and then we generated the stable HDAC7-overexpressing EC109 cells and HDAC7-knockdown EC9706 cells. Then, we established the EC109 cell xenograft in athymic nude mice, and found the xenograft tumors of the HDAC7-overexpressing (OE) group weighed higher than that of the NC group (*P* = 0.0123, Fig. 2e). Moreover, both colony formation and EdU incorporation assays indicated that HDAC7 overexpression significantly enhanced the EC109 cell growth, whereas HDAC7 knockdown decreased the proliferative ability of EC9706 cells relatively compared with the control group (*P* < 0.05, Fig. 2f). Interestingly, Western blotting analysis showed that HDAC7 overexpression significantly increased the c-Myc expression and decreased the p21/p27 levels in EC109 cells, but loss of HDAC7 reversed expression of these proteins in EC9706 cells, suggesting HDAC7 positively regulates the c-Myc/p21/p27 pathway (Fig. 2g). To summarize, above findings demonstrate that high HDAC7 expression predicts poor prognosis of ESCC patients, and HDAC7 works as an oncogene via activating c-Myc signaling thus enhancing ESCC cells proliferation.

### HDAC7 mediates the anti-proliferative action of melatonin on ESCC cells

To detect whether HDAC7 mediated the anti-proliferative action of melatonin on ESCC cells, we performed the colony formation and EdU incorporation assays in vitro. Compared with the single melatonin treatment group, the melatonin-induced inhibition of cell growth and colony formation was partially rescued by HDAC7 overexpression in melatonin-treated EC109 cells, but markedly sensitized by HDAC7 knockdown in the melatonin-treated EC9706 cells (Fig. 2f,  *P* < 0.05). Furthermore, Western blotting analysis showed that the melatonin-induced downregulation of c-Myc and upregulation of p21 and p27 levels was also reversed by HDAC7 overexpression whereas enhanced by HDAC7 knockdown in the melatonin-treated ESCC cells (Fig. 2g). Collectively, these results indicated that HDAC7 inhibition mediated anti-proliferative role of melatonin against ESCC cells.

### HDAC7 and c-Myc form a positive feedback loop to enhance ESCC growth

**Fig. 3:**
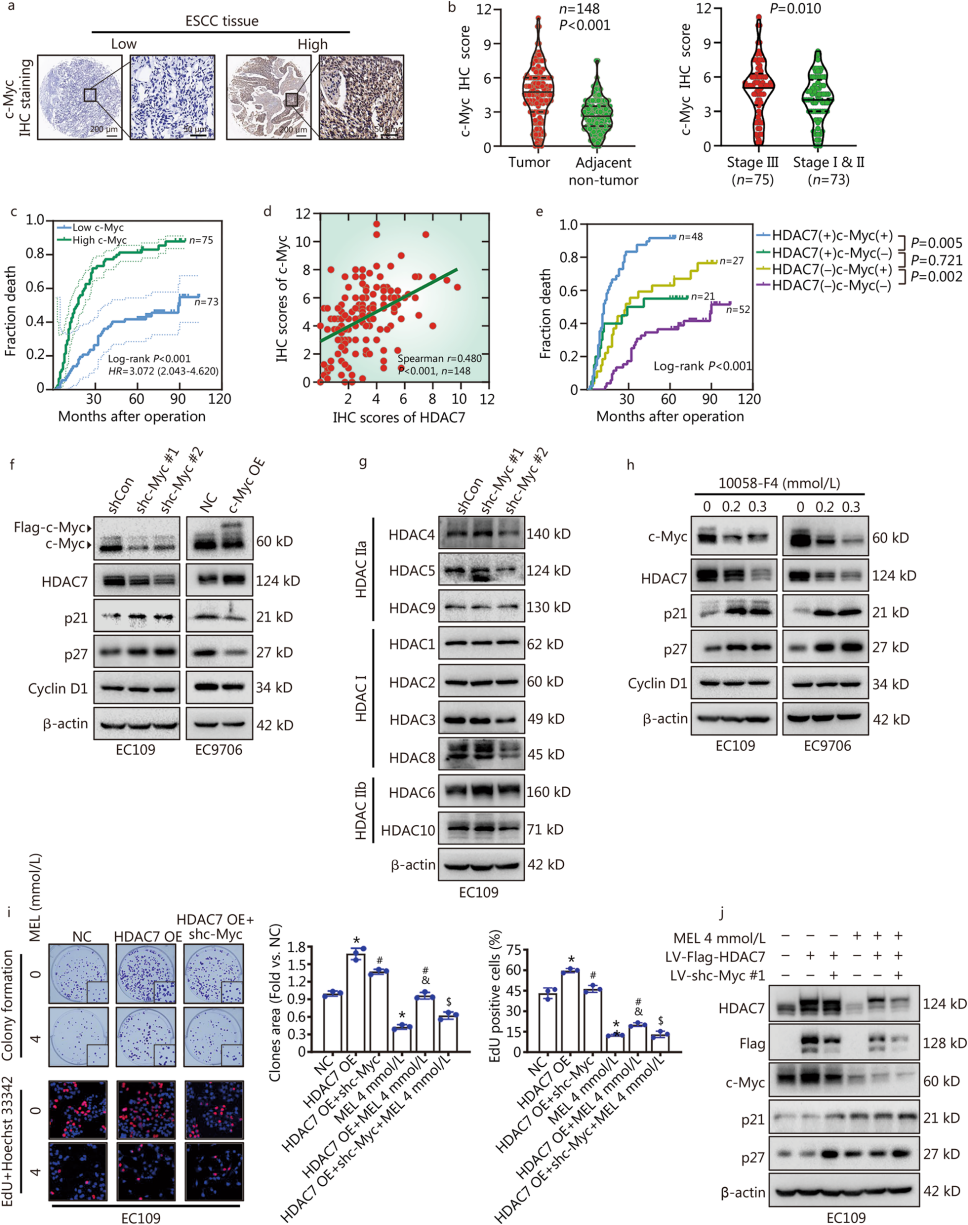
HDAC7-c-Myc positive feedback loop to promote ESCC growth and involves in the anti-proliferative action of melatonin on ESCC cells. **a** Representative IHC images for c-Myc expression in ESCC tissues. Scale bar, 200 μm and 50 μm (inset), respectively. **b** Statistical analysis of c-Myc expression in 148 ESCC patients through IHC staining. **c** Kaplan–Meier survival analysis by high/low c-Myc levels of the 148 ESCC patients based on the microarray tissue IHC results. **d** Spearman correlation analyses of HDAC7 and c-Myc expression in the ESCC tissues. **e** Kaplan–Meier analysis of the association between overall survival and the expression of HDAC7 and c-Myc on 148 ESCC patients. **f** Representative Western blotting results of c-Myc, HDAC7, p21, p27 and Cyclin D1. **g** Representative Western blotting results of HDACs in the EC109 cells with c-Myc knockdown. **h** Representative Western blotting results of c-Myc, HDAC7, p21, p27 and Cyclin D1 in the EC109 and EC9706 cells with 10058-F4 treatment. **i** Representative images and results of colony formation and EdU incorporation assays. **j** Co-treatment of melatonin, LV-Flag-HDAC7 and LV-shc-Myc #1 was applied to EC109 cells. Representative Western blotting results of HDAC7, Flag-HDAC7, c-Myc, p21 and p27 were shown. ^*^P < 0.05 vs. the NC group, ^#^*P* < 0.05 vs*.* the HDAC7 OE group, ^&^*P* < 0.05 vs*.* the MEL 4 mmol/L group, ^$^*P* < 0.05 vs*.* the HDAC7 OE + MEL 4 mmol/L group. HDAC7 histone deacetylase 7, ESCC esophageal squamous cell carcinoma, OE overexpression, LV lentivirus, MEL melatonin, IHC immunohistochemistry

To further evaluate the clinical relevance of the c-Myc and HDAC7 expression in ESCC tissues, IHC staining was applied to detect the c-Myc level in the tissue microarrays (Fig. 3a). The expression of c-Myc was higher in the ESCC than their paired normal tissues (*P* < 0.001, Fig. 3b), and ESCC tissues from the high pTNM stage (III) had higher c-Myc expression than those with low pTNM stage (I & II) (*P* = 0.010, Fig. 3b). Moreover, the Cox and Kaplan–Meier survival analyses respectively indicated that c-Myc expression was an independent prognostic factor for ESCC patients (*HR* = 2.233, 95% CI 1.376 – 3.625, *P* = 0.001, Table 2), and negatively correlated with ESCC overall survival (Log-rank *P* < 0.001, Fig. 3c). Later, to further clarify the possible correlation between HDAC7 and c-Myc expression in the ESCC tissues, the spearman correlation analyses showed that HDAC7 expression positively correlated c-Myc level (Spearman *r* = 0.480, *P* < 0.001, Fig. 3d). Then patients were divided into four groups according to the HDAC7 and c-Myc levels. Importantly, the Kaplan–Meier analyses suggested that patients with high HDAC7/c-Myc had the worst prognosis, whereas ESCC with low HDAC7/low c-Myc showed the best outcome (Fig. 3e).

In order to confirm the function of c-Myc on ESCC proliferation, we tested the c-Myc level of the ESCC cell lines (Additional file 1: Fig. S3a), and then we built the stable c-Myc-knockdown EC109 cells and c-Myc-overexpressing EC9706 cells (Fig. 3f). Surprisingly, c-Myc knockdown could remarkably decrease the expression of HDAC7 instead of other HDACs, and increase the downstream p21/p27 levels in the EC109 cells (Fig. 3f, g). Conversely, c-Myc overexpression markedly promoted HDAC7 and inhibited p21/p27 expression in the EC9706 cells (Fig. 3f). To make further conformation, both the EC109 and EC9706 cells were treated with the c-Myc-specific inhibitor 10058-F4, then c-Myc inhibition significantly downregulated the HDAC7 level and enhanced the p21/p27 expression (Fig. 3h). Altogether, these results revealed that HDAC7 and c-Myc could enhance each other’s expression, thus forming a positive feedback loop.

### Melatonin decreases ESCC cell proliferation via inhibiting HDAC7-c-Myc positive feedback loop

To verify the impact of the HDAC7-c-Myc signaling on mediating ESCC proliferation and melatonin’s anti-proliferative activity, we examined the actions of knocking down c-Myc via further infecting shc-Myc lentivirus on the EC109 cells stably overexpressing HDAC7. c-Myc knockdown partially reversed the HDAC7 overexpression-induced cell growth promotion, and further decreased the c-Myc and ectopic HDAC7 protein levels and increased the p21/p27 expression in the EC109 cells with HDAC7 overexpression (Fig. 3i, j). In addition, compared with the HDAC7 OE + MEL 4 mmol/L group, c-Myc knockdown significantly enhanced the melatonin’s actions of anti-proliferation, HDAC7/c-Myc inhibition and p21/p27 upregulation on the HDAC7 OE EC109 cells (Fig. 3i, j). Given the above, we suggest that melatonin treatment hinders ESCC cell growth via the inhibition of the HDAC7-c-Myc positive feedback loop.

### Melatonin interrupts HDAC7-mediated β-catenin deacetylation and nuclear transport

**Fig. 4:**
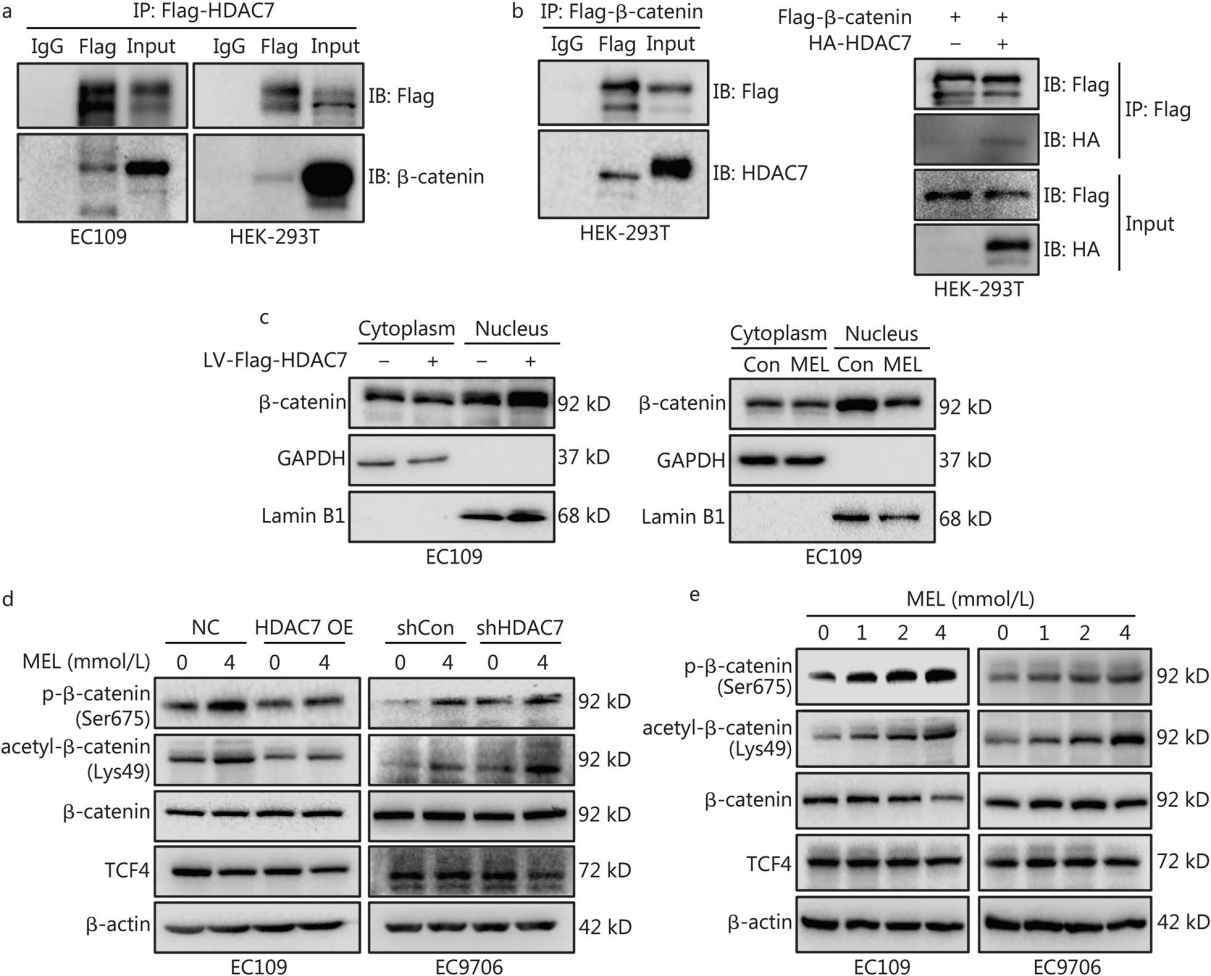
HDAC7 promotes β-catenin deacetylation, dephosphorylation and nuclear transport, whereas melatonin interrupts above actions. **a** and **b** Co-IP analysis was performed to determine the interaction between HDAC7 and β-catenin in both EC109 and HEK-293T cells. HDAC7 or β-catenin proteins were respectively immunoprecipitated by the anti-Flag antibody followed by target proteins detection using Western blotting analyses. **c** Cytosolic fractionation and immunoblot assays were applied to detect β-catenin nuclear import after overexpressing HDAC7 by infecting LV-Flag-HDAC7 or melatonin treatment in EC109 cells; GAPDH and Lamin B1 were respectively cytoplasmic and nucleus loading controls. **d** and **e** Melatonin treatment for 48 h enhanced β-catenin phosphorylation (Ser675) and acetylation (Lys49) without affecting total β-catenin and TCF4 levels, whereas HDAC7 overexpression or downregulation could partially hinder or enhance the above melatonin’s actions. Representative Western blotting results of phosphorylated β-catenin (Ser675), acetylated β-catenin (Lys49), β-catenin and TCF4 were shown. HDAC7 histone deacetylase 7, LV lentivirus, MEL melatonin, TCF4 transcription factor 4, OE overexpression

To explore the molecular mechanisms of HDAC7 enhancing c-Myc expression, co-IP was conducted and validated that HDAC7 could directly bind β-catenin in both EC109 and HEK-293T cells (Fig. 4a, b). β-catenin is the central player of the Wnt signaling cascade, and the activated (unphosphorylated) β-catenin accumulates, transports from the cytoplasm into the nucleus and then binds to transcription factor 4 (TCF4), thus promoting the expression of target genes such as *c-Myc* [17]. Therefore, we hypothesized that β-catenin is a key factor in mediating HDAC7-dependent c-Myc expression. By the cytosolic and nuclear fractionation, we found HDAC7 overexpression promoted β-catenin nuclear import (Fig. 4c, left panel), whereas HDAC7 inhibition by melatonin markedly impaired the β-catenin nuclear redistribution (Fig. 4c, right panel). Previous literatures reported that β-catenin deacetylation (Lys49) could hinder the phosphorylation of β-catenin thus activating β-catenin [17, 21]. Consistently, in this study, ectopic HDAC7 expression markedly downregulated the β-catenin acetylation (Lys49) and phosphorylation (Ser675) levels; while HDAC7 knockdown promoted β-catenin acetylation and phosphorylation in ESCC cells (Fig. 4d). To summarize, the above findings demonstrate that HDAC7-induced β-catenin deacetylation/dephosphorylation leads to β-catenin nuclear import.

Furthermore, to verify the actions of melatonin on β-catenin acetylation and phosphorylation, Western blotting analyses showed the dose-dependent increase of the β-catenin acetylation and phosphorylation after melatonin exposure on ESCC cells (Fig. 4e). In particular, we noticed that both HDAC7 expression changes and melatonin treatment had no significant effect on the TCF4 and total β-catenin expression levels (Fig. 4d, e). Notably, HDAC7 overexpression could hinder the melatonin-induced β-catenin acetylation and phosphorylation whereas HDAC7 knockdown thus enhancing the above melatonin’s actions (Fig. 4d). Therefore, these results indicated that melatonin decreased β-catenin nuclear distribution via inhibiting the HDAC7-dependent β-catenin deacetylation/dephosphorylation.

### Melatonin enhances the anti-proliferative action of β-catenin or HDACs inhibitor on ESCC cells

**Fig. 5:**
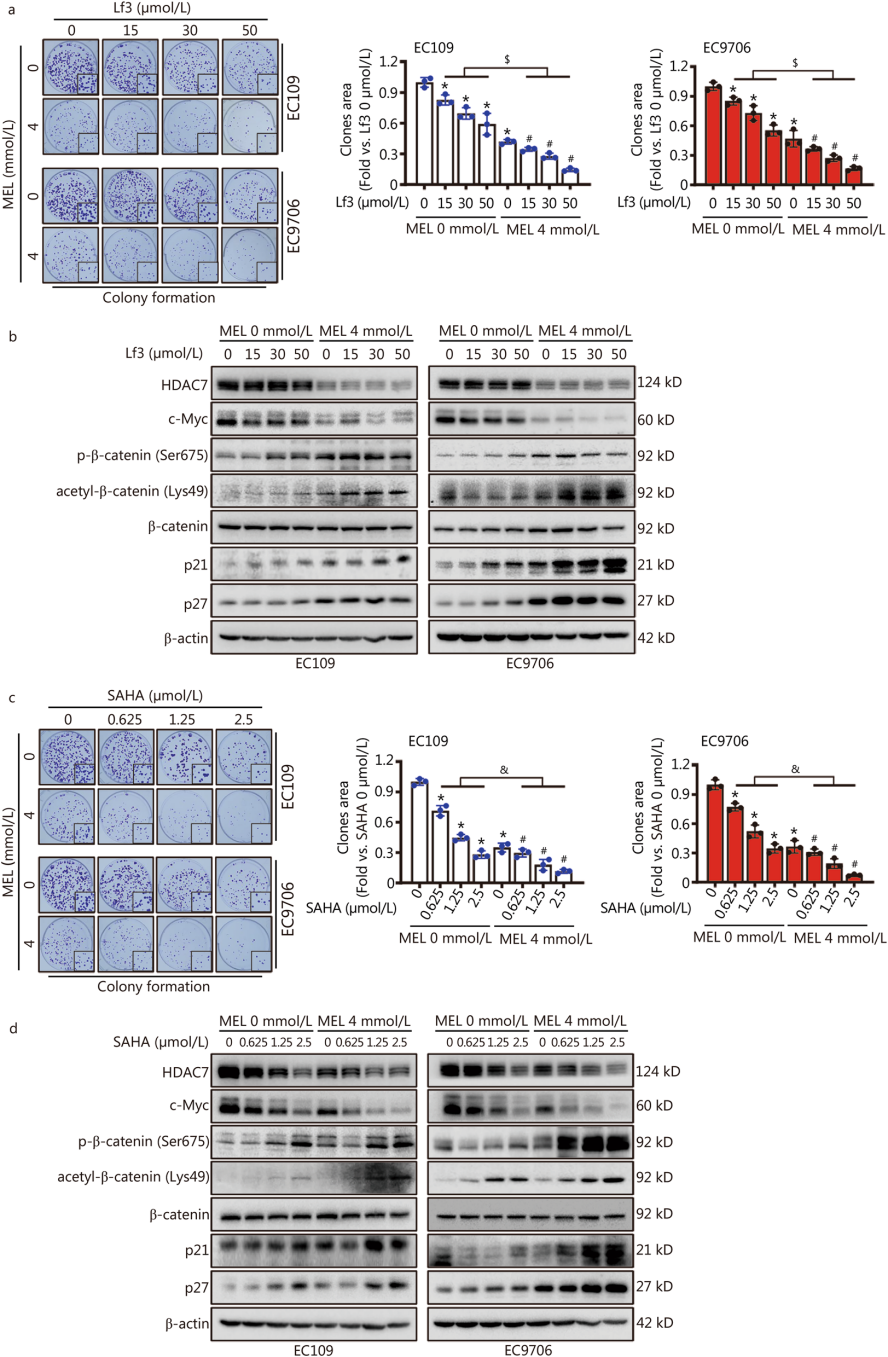
Lf3 and SAHA inhibits ESCC cell growth and β-catenin/c-Myc signaling, and melatonin co-treatment further enhances these actions. **a** and **c** Representative images and results of colony formation assay with Lf3 + melatonin or SAHA + melatonin pretreatments for 48 h in the EC109 and EC9706 cells. **b** and **d** Co-treatment of Lf3 + melatonin or SAHA + melatonin was applied to EC109 and EC9706 cells for 48 h. Representative Western blotting results of HDAC7, c-Myc, phosphorylated β-catenin (Ser675), acetylated β-catenin (Lys49), β-catenin, p21 and p27 were shown. ^*^P < 0.05 vs. the Lf3 0 μmol/L group + MEL 0 mmol/L group, ^#^*P* < 0.05 vs. the Lf3 0 μmol/L group + MEL 4 mmol/L group, ^$^*P* < 0.05 vs. the only Lf3 treatment groups, ^&^*P* < 0.05 vs. the only SAHA treatment groups. HDAC7 histone deacetylase 7, MEL melatonin

To further understand the role of β-catenin on mediating HDAC7-induced c-Myc upregulation, the specific β-catenin activity inhibitor Lf3 was applied to the ESCC cells, and Lf3 treatment dose-dependently decreased the ESCC cell proliferation (*P* < 0.05, Fig. 5a). In particular, Lf3 treatment just contribute to c-Myc downregulation and p21/p27 upregulation without changing the HDAC7, acetylated/phosphorylated β-catenin and total β-catenin protein levels (Fig. 5b). After HDAC7 inhibition by melatonin co-treatment, the anti-proliferative action of Lf3 on ESCC cells was markedly enhanced (*P* < 0.05, Fig. 5a). In addition, compared with the single Lf3 treatment groups, melatonin and Lf3 co-treatment further decreased the HDAC7 and c-Myc expression, and markedly increased the acetylated/phosphorylated β-catenin and p21/p27 protein levels (Fig. 5b). Therefore, these results suggested that the activity of β-catenin could be activated by HDAC7 thus motivating the c-Myc signaling, and the above signaling pathway could be inhibited by melatonin treatment.

SAHA is a pan-HDACs inhibitor with anticancer activities [31]. In Fig. 5c, SAHA treatment significantly inhibited the ESCC cells colony formation in a dose-dependent manner (*P* < 0.05). Meanwhile, Western blotting analysis further showed the dose-dependent decrease of HDAC7 and c-Myc expression, and increase of β-catenin acetylation/phosphorylation, p21 and p27 protein levels (Fig. 5d). Intriguingly, melatonin co-treatment significantly enhanced the anti-proliferative role of SAHA on ESCC cells (*P* < 0.05, Fig. 5c). Moreover, compared with the SAHA alone treatment group, co-treatment of melatonin and SAHA markedly inhibited the HDAC7 and c-Myc expression, and upregulated the β-catenin acetylation/phosphorylation, p21 and p27 protein levels (Fig. 5d). Accordingly, above results indicated that melatonin enhances the anti-proliferative action of β-catenin or HDACs inhibitor on ESCC cells via hindering the HDAC7-β-catenin-c-Myc signaling.

### USP10 deubiquitinates and stabilizes HDAC7 protein in ESCC cells

**Fig. 6:**
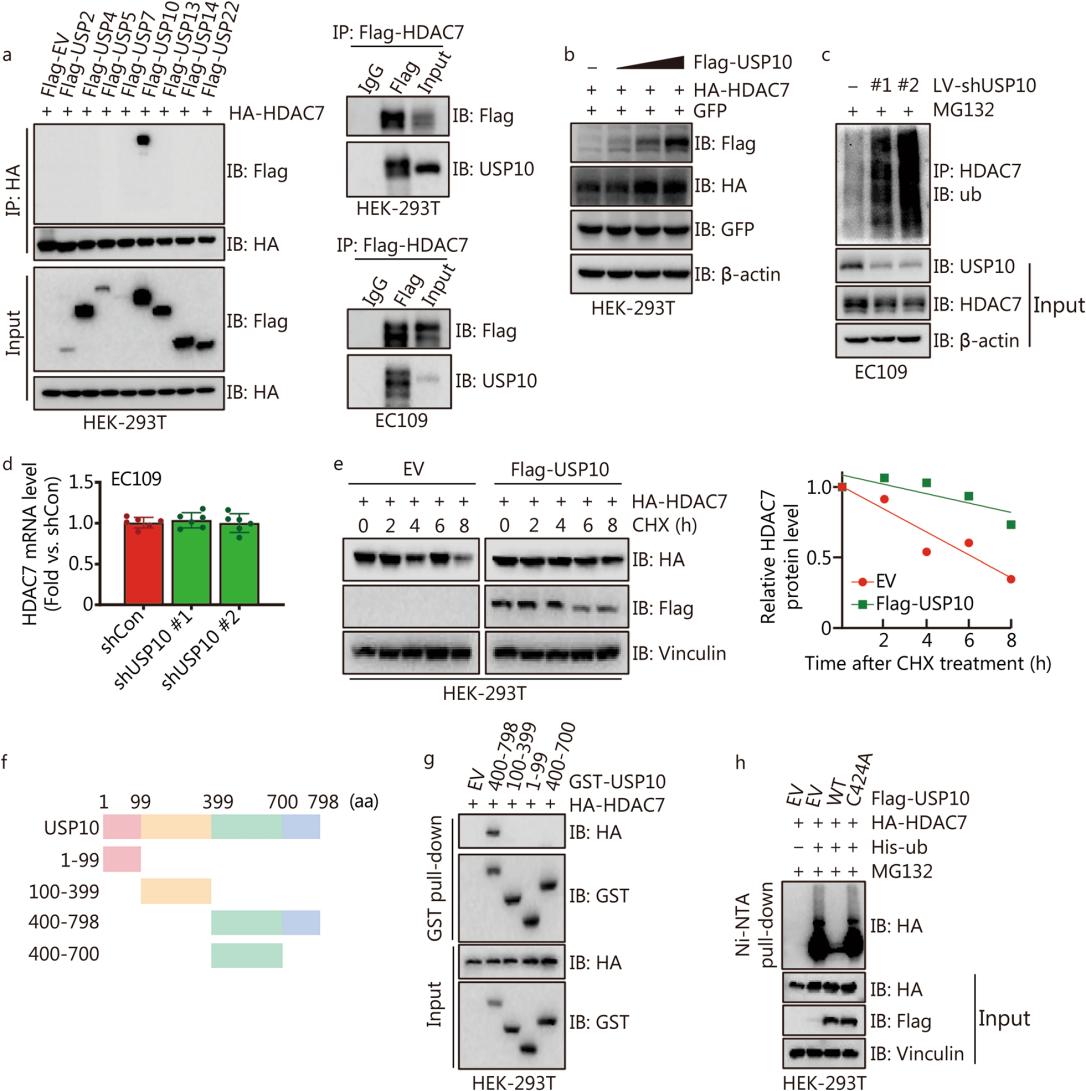
USP10 deubiquitinates and stabilizes HDAC7 protein in ESCC cells. **a** Indicated Flag-tagged USPs and HA-HDAC7 were transfected into HEK-293T cells, and Flag-tagged HDAC7 was respectively transfected into HEK-293T and EC109 cells, then co-IP analysis was performed to determine the interaction between HDAC7 and USP10 in vitro by Western blotting analyses. **b** Immunoblot analyses of whole cell lyses derived from HEK-293T cells transfected with indicated Flag-USP10, HA-HDAC7 and GFP plasmids. **c** Immunoblot analyses of input and HDAC7 immunoprecipitated from EC109 cells with USP10 knocking down, and treated with 10 μmol/L MG132 overnight before harvesting. **d** Result of HDAC7 mRNA levels in the EC109 cells with USP10 knocking down. **e** HEK-293T cells were transfected with indicated Flag-USP10 and HA-HDAC7 plasmids, then cells were treated with CHX at indicated time points. The HDAC7 protein level was quantified by the ImageJ software and plotted. **f** Overview of USP10 protein structures. **g** HEK-293T cells transfected with the indicated constructs were subjected to pull down with anti-HA or anti-GST. **h** HDAC7 ubiquitylation was analyzed in HEK-293T cells transfected with USP10/WT or USP10/C424A. USPs ubiquitin-specific proteases, HDAC7 histone deacetylase 7, ub ubiquitin, EV empty vector, WT wild type, CHX cycloheximide, USP10 ubiquitin-specific peptidase 10

The protein level and stability of HDAC7 can be mainly regulated by ubiquitination/deubiquitination [32]. It is well documented that the activity of ubiquitin ligases can be reversed by ubiquitin-specific proteases (USPs) [22]. To screen the potential deubiquitinating enzymes of HDAC7, a panel of Flag-tagged USPs and HA-tagged HDAC7 were transfected into HEK-293T cells. Immunoblotting assays showed HA-HDAC7 could only be detected on anti-Flag beads conjugated with USP10 (Fig. 6a). We double confirmed that Flag-HDAC7 physically interacted with endogenous USP10 in both HEK-293T and EC109 cells (Fig. 6a). Moreover, ectopic expression of Flag-USP10 resulted in dose-dependent elevation of HA-HDAC7 in HEK-293T cells (Fig. 6b), whereas USP10 knockdown markedly increased HDAC7 polyubiquitination and decreased the HDAC7 protein expression without influencing the HDAC7 mRNA levels in EC109 cells (Fig. 6c, d). Since USP10 acts as the deubiquitinase to remove ubiquitination from its substrates, we hypothesized that USP10 could stabilize HDAC7 protein. The CHX assay indicated that USP10 overexpression prolonged the half-life of HDAC7 (Fig. 6e). Next, to gain more insight into the USP10-HDAC7 interaction, a series of GST-tagged truncated mutants of USP10 and HA-tagged HDAC7 were co-transfected, and the C-terminal region (701–798 aa) of USP10 was determined as the binding region with HDAC7 (Fig. 6f, g). Next, the wild-type USP10 (USP10/WT) and catalytically inactive USP10 mutant (USP10/C424A) were generated [24]. Ubiquitylation assay showed that the ectopic USP10/WT expression markedly deubiquitinated HDAC7, while C424A mutant disturbed USP10’s above deubiquitinating actions (Fig. 6h). Taken together, our results demonstrate that USP10 interacts with HDAC7 and stabilize HDAC7 protein through promoting HDAC7 deubiquitination.

### Correlation of USP10, HDAC7 and c-Myc expression with ESCC prognosis

**Fig. 7:**
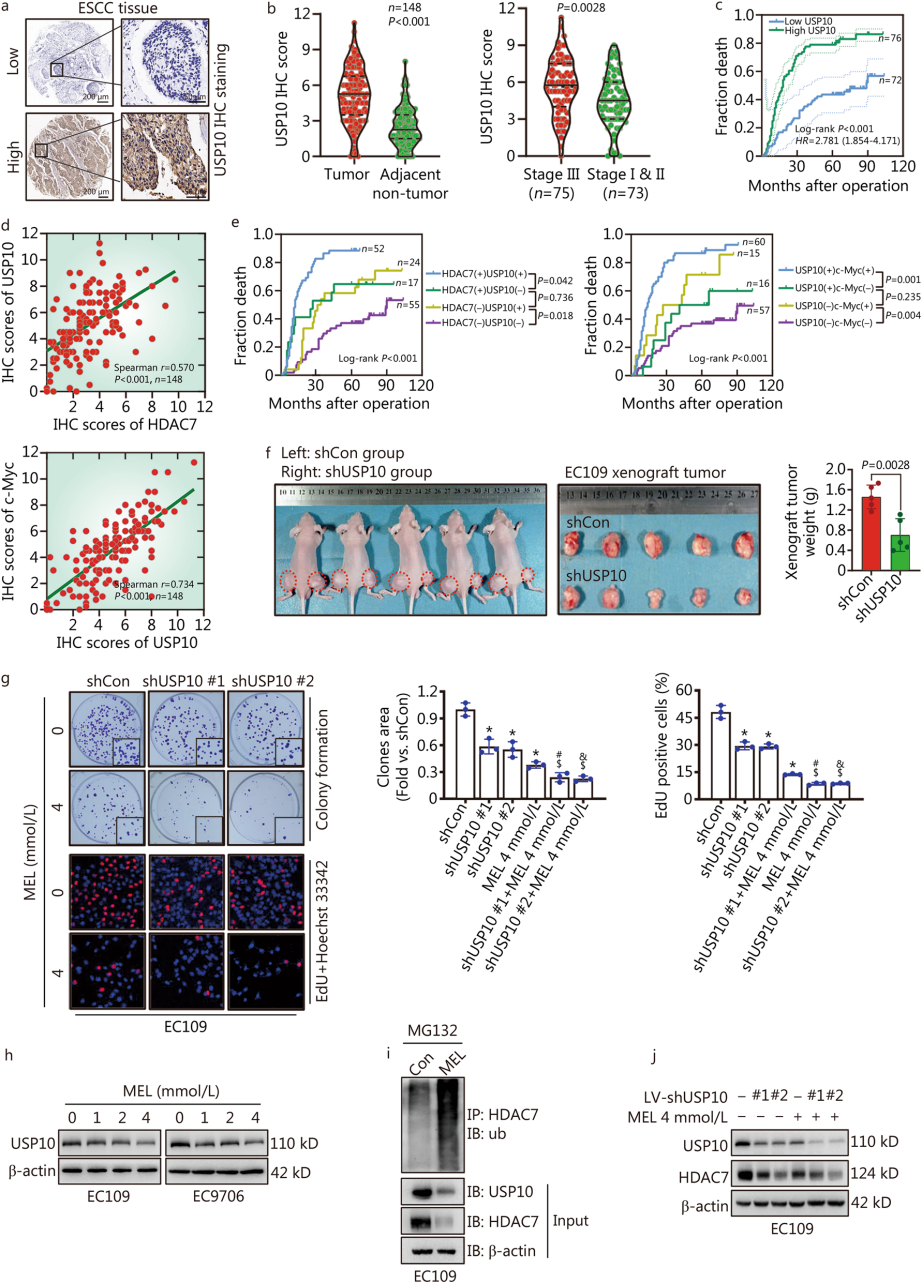
Melatonin inhibits USP10-mediated HDAC7 deubiquitination thus inducing HDAC7 proteolysis. **a** Representative IHC images for USP10 expression in ESCC tissues. Scale bar, 200 μm and 50 μm (inset), respectively. **b** Statistical analysis of USP10 expression in 148 ESCC patients through IHC staining. **c** Kaplan–Meier survival analysis by high/low USP10 levels of the 148 ESCC patients based on the microarray tissue IHC results. **d** The spearman correlation analyses of USP10, HDAC7 and c-Myc expression in the ESCC tissues. **e** Kaplan–Meier analysis of the association between overall survival and the expression of USP10, HDAC7 and c-Myc in 148 ESCC patients. **f** Photographs and results showing xenograft tumor morphologies and weights in each group after subcutaneously injection of shCon or shUSP10 EC109 cells, respectively. **g** Representative images and results of colony formation and EdU incorporation assays in the EC109 cells with melatonin and USP10 knockdown treatment. **h** Western blotting results showed melatonin treatment for 48 h decreased USP10 levels in ESCC cells. **i** Immunoblot analyses of input and HDAC7 immunoprecipitated from EC109 cells with melatonin exposure and treated with 10 μmol/L MG132 overnight before harvesting. **j** Representative western blotting results of USP10 and HDAC7 in the EC109 cells with melatonin and USP10 knockdown treatment. ^*^P < 0.05 vs. the shCon group, ^#^*P* < 0.05 vs. the shUSP10 #1 group, ^&^*P* < 0.05 vs. the USP10 #2 group, ^$^*P* < 0.05 vs. the MEL 4 mmol/L group. USP10 ubiquitin-specific protease 10, HDAC7 histone deacetylase 7, ESCC esophageal squamous cell carcinoma, ub ubiquitin, MEL melatonin, LV lentivirus, IHC immunohistochemistry, USP10 ubiquitin-specific peptidase 10

To evaluate the clinical importance of the USP10-HDAC7-c-Myc axis and determine their correlation in ESCC, we further detected the USP10 expression in tissue microarrays via IHC staining (Fig. 7a). USP10 was highly expressed in the ESCC than adjacent normal tissues (*P* < 0.001, Fig. 7b), and ESCC tissues from the patients with high pTNM stage (III) expressed USP10 higher than those from patients with low pTNM stage (I & II) (*P* = 0.0028, Fig. 7b). Moreover, both the Kaplan–Meier and Cox survival analyses suggested that USP10 was a negative prognostic factor for ESCC patients (*HR* = 1.725, 95% CI 1.032–2.885, *P* = 0.038, Table 2), and higher USP10 level predicted shorter overall survival (Log-rank *P* < 0.001, Fig. 7c). The possible correlation between the expression of USP10 and HDAC7/c-Myc in the ESCC tissues were further evaluated. The spearman correlation analyses showed that the USP10 positively correlated with HDAC7 and c-Myc (Spearman *r* = 0.570, *P* < 0.001, Fig. 7d). Then patients were divided into four groups according to the levels of USP10, HDAC7 and c-Myc expression. Importantly, the Kaplan–Meier analyses showed that patients with high coexpression of USP10 and HDAC7 or of USP10 and c-Myc had the worst prognosis; whereas ESCC with low USP10/low HDAC7, and low USP10/low c-Myc showed the best prognosis among the groups (Fig. 7e).

### Melatonin inhibits USP10-mediated HDAC7 deubiquitination and stability

USP10 was reported to promote the hepatocellular and lung cancer cells proliferation [22, 24], but its function on ESCC remains to be elucidated. To investigate the action of USP10 on ESCC cells proliferation, we performed the colony formation, EdU incorporation and tumor xenograft formation assays. Both the in vivo and in vitro results indicated that USP10 knockdown significantly inhibited the growth of EC109 cells (*P* < 0.05, Fig. 7f, g). Notably, melatonin treatment dose-dependently decreased the USP10 levels and markedly enhanced the ubiquitination of HDAC7 protein in the EC109 cells (Fig. 7h, i). Moreover, compared with the USP10 knockdown group, melatonin treatment further intensified the USP10 knockdown-induced growth inhibition and the decrease of USP10 and HDAC7 levels (Fig. 7g, j). In addition, we further tested whether HDAC7, β-catenin and c-Myc influence the USP10 expression in ESCC cells. Interestingly, none of the HDAC7 overexpression/knockdown, β-catenin activity inhibition by Lf3, c-Myc overexpression/knockdown, and c-Myc inhibition by 10058-F4 had significant influence on the USP10 expression in the EC109 and EC9706 cells (Additional file 1: Fig. S3b-f). Overall, these results demonstrate that melatonin-caused HDAC7 downregulation may also attribute to HDAC7 protein degradation via inhibiting USP10 expression.

## Discussion

Previous studies revealed melatonin has significant inhibitory effects on numerous cancers [5–9]. Melatonin exerted anticancer actions in almost every stage of tumor generation and development through the joint effect of multiple functions, such as inhibiting proliferation, angiogenesis and metastasis, affecting aerobic metabolism, and enhancing chemotherapy efficiency [5–11]. Our previous study reported that melatonin treatment could significantly mitigate lung cancer cell growth via inhibiting the HDAC9 signaling [1]. Interestingly, the present experimental data indicated that melatonin dose-dependently suppressed the ESCC cell proliferation and markedly downregulated the HDAC7 mRNA and protein levels without influencing other HDACs expression in the ESCC cells, suggesting that melatonin works as a potential specific HDAC7 inhibitor for ESCC treatment.

HDAC7 expression is dysregulated in many cancers, and high HDAC7 level was associated with poor prognosis of lung and gastric cancers [15, 19]. Moreover, experimental studies have proven that HDAC7 overexpression could promote the growth of lung cancer [15], breast cancer [16], glioma [17], and nasopharyngeal carcinomas cells [33]. However, the prognostic role and function of HDAC7 on the ESCC are still unclear. Our current study analyzed the HDAC7 expression in ESCC and adjacent normal tissues via IHC staining. We reported a remarkable high HDAC7 level in ESCC than that of normal tissues, and survival analyses indicated that high HDAC7 expression was an independent negative prognostic factor for ESCC patients. Later, both the in vitro and in vivo studies further confirmed ectopic HDAC7 expression markedly enhanced ESCC cells growth. Thus, these results strongly demonstrate that HDAC7 functions as an oncogene on ESCC.

Recent researches revealed that oncogenic actions of HDAC7 are dependent on the c-Myc amplification [18, 32], since c-Myc helps tumor cells escape from cellular senescence process and promotes tumor cell growth via inhibiting p21/p27 expression thus accelerating G1–S cell cycle transition [18, 30]. Zhu et al. [18] found HDAC7 silencing blocked cell cycle progression via inhibiting c-Myc expression in the Hela and MCF-7 cells. Consistently, we found HDAC7 overexpression promoted ESCC cell growth and c-Myc expression and decreased the p21/p27 levels. Surprisingly, c-Myc knockdown could uniquely decrease the expression of HDAC7 instead of other HDACs, and partially hindered the HDAC7 overexpression-induced ESCC cell proliferation. These results further illustrated that HDAC7 and c-Myc formed a positive feedback loop to enhance ESCC cell growth. Melatonin has been reported as a potential HDAC IIa inhibitor for lung cancer cells that could disturb c-Myc expression in the brain cancer stem cells [1, 34]. In the current study, melatonin treatment specifically suppressed HDAC7 and c-Myc expression in ESCC cells. Later, we found the melatonin-induced ESCC cell growth suppression was partially hindered by HDAC7 overexpression while was then rescued by further c-Myc silencing. Given the above, we suggest that melatonin treatment inhibits ESCC cell growth via suppressing the HDAC7-c-Myc positive feedback loop.

To further reveal the molecular mechanism of HDAC7-enhanced c-Myc activation, we focused on the β-catenin signaling pathway. Activated (unphosphorylated) β-catenin accumulates, transports from the cytoplasm into the nucleus and forms complexes with the TCF/LEF transcription factors then activating target genes such as *c-Myc* and *CCND1* [17, 21]. In this study, we found HDAC7 could bind to β-catenin and deacetylate β-catenin Lys49 in the ESCC cells. Previous studies demonstrated that β-catenin Lys49 deacetylation led to β-catenin dephosphorylation thus promoting β-catenin import and activation [17, 21]. Consistently, our data further confirmed that HDAC7 overexpression also hindered β-catenin phosphorylation and promoted its nuclear import. Moreover, HDAC7 inhibition by melatonin treatment markedly impaired the β-catenin nuclear redistribution and activation, whereas HDAC7 overexpression partially reversed the melatonin’s above functions. Accordingly, these results further indicate that melatonin-caused c-Myc suppression is regulated by hindering HDAC7-dependent β-catenin activation.

Ubiquitination/deubiquitination involves balancing the protein stability and ubiquitin-dependent protein degradation [35]. After screening the potential deubiquitinating enzymes of HDAC7, we first reported deubiquitinase USP10 could bind and stabilize HDAC7 protein in ESCC cells. USP10 exerts diverse functions on tumor progression, and the oncogenic or oncostatic function of USP10 was largely dictated by its substrate(s) in the specific cellular context of cancer models [22, 25, 36, 37]. Our IHC staining analysis reported that high USP10 expression may be related to poor ESCC survival prognosis. In the ESCC cells, we further found USP10 promotes EC109 cells proliferation via deubiquitinating and stabilizing HDAC7. Therefore, promotion of USP10-HDAC7 axis drives ESCC cell growth. Interestingly, we also found melatonin treatment markedly decreased the USP10 expression and enhanced the HDAC7 protein ubiquitination in ESCC cells. Accordingly, except for directly inhibiting HDAC7 transcription, melatonin-caused HDAC7 protein degradation via inhibiting USP10 expression may also attribute to HDAC7 downregulation.

Numerous studies on melatonin as an oncostatic agent have been published over the past 50 years, and many researches from cancer cell lines, animal models, and xenografts in rodents have revealed that melatonin inhibits various cancers proliferation by mediating a large number of molecular pathways [8, 38]. Our current study just confirmed melatonin exerts anti-proliferative actions on ESCC cells by inhibiting HDAC7/β-catenin/c-Myc positive feedback loop and USP10/HDAC7 signaling in vitro. Future in vivo experiments are needed to verify the above results. Additionally, melatonin synergizes with chemotherapy [8, 39, 40], and our experimental data indicated melatonin enhances the anti-proliferative action of β-catenin or HDACs inhibitors on ESCC cells. Future research directions should include the practical applications of melatonin based on pre-clinical and clinical studies, and determining in more detail and with more accuracy the molecular pharmacological mechanisms of melatonin in cancer treatment.

## Conclusions

**Fig. 8:**
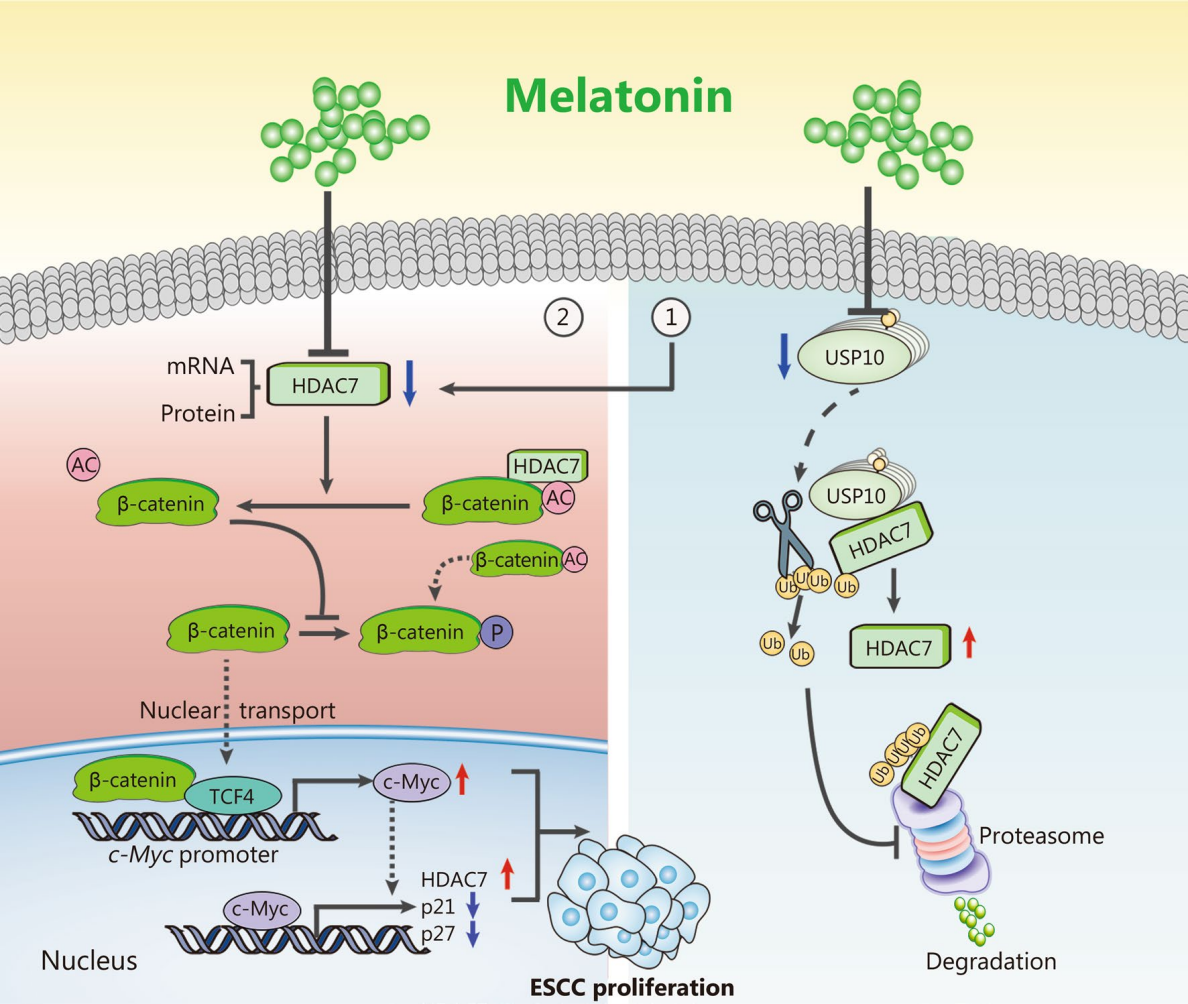
Schematic diagram about the molecular mechanism of melatonin inhibiting ESCC cell growth. ① USP10 deubiquitinates and stabilizes HDAC7 protein in the ESCC cells. Moreover, melatonin downregulates HDAC7 by both decreasing HDAC7 transcription and reducing protein stability through USP10 inhibition. ② HDAC7 physically deacetylates β-catenin Lys49, thus hindering β-catenin phosphorylation and subsequently activating target *c-Myc* gene expression. Interestingly, HDAC7/β-catenin/c-Myc could form a positive feedback loop to enhance ESCC cell growth, and melatonin works as a potent HDAC7 inhibitor thus prohibiting ESCC proliferation. USP10 ubiquitin-specific protease 10, HDAC7 histone deacetylase 7, ESCC esophageal squamous cell carcinoma

Collectively, our basic data verified that HDAC7/β-catenin/c-Myc formed positive feedback loop to enhance ESCC cell proliferation, and oncoprotein HDAC7 could be stabilized by USP10. Moreover, we further revealed that melatonin functioned as a potential HDAC7 inhibitor to suppress ESCC growth via decreasing HDAC7 transcription and inducing ubiquitin-dependent HDAC7 protein degradation by USP10 inhibition (Fig. 8). Therefore, above experimental results indicate that targeting the HDAC7/β-catenin/c-Myc axis and USP10/HDAC7 pathway, and combining it with melatonin may be a novel strategy for anti-ESCC therapy.

## Supplementary Information

**Additional file 1:** **Table S1.** Detailed information of the primary antibodies. **Fig. S1**. Criteria of the IHC staining scores. **Fig. S2**. Melatonin treatment for 48 h did not affect the expression of the listed cell cycle-related proteins in the EC109 and EC9706 cells. Representative Western blotting results of CDK1, CDK2, CDK4, CDK6, Cyclin A2, Cyclin B1 and Cyclin E1 were shown. **Fig. S3**. USP10 expression is not regulated by the HDAC7/β-catenin/c-Myc pathway.

## Data Availability

Data collected and analyzed for the study are available from the corresponding author upon reasonable request.

## Abbreviations

CHX: Cycloheximide
CDK: Cyclin-dependent kinase
EdU: 5-Ethynyl-2′-deoxyuridine
ESCC: Esophageal squamous cell carcinoma
EV: Empty vector
HDAC7: Histone deacetylase 7
LV: Lentivirus
IHC: Immunohistochemistry
IP: Immunoprecipitation
MEL: Melatonin
OE: Overexpression
STAT3: Signal transducer and activator of transcription 3
TCF4: Transcription factor 4
ub: Ubiquitin
USP10: Ubiquitin-specific peptidase 10
WT: Wild type
