# Supplementary material for: Melatonin inhibits ESCC tumor growth by mitigating the HDAC7/β-catenin/c-Myc positive feedback loop and suppressing the USP10-maintained HDAC7 protein stability
Source: Mil Med Res. 2022 Sep 27;9:54. doi: 10.1186/s40779-022-00412-0 (PMC9513894; doi:10.1186/s40779-022-00412-0)
Supplement: Supplementary file 1 — Additional file 1: Table S1. Detailed information of the primary antibodies. Fig. S1. Criteria of the IHC staining scores. Fig. S2. Melatonin treatment for 48 h did not affect the expression of the listed cell cycle-related proteins in the EC109 and EC9706 cells. Representative Western blotting results of CDK1, CDK2, CDK4, CDK6, Cyclin A2, Cyclin B1 and Cyclin E1 were shown. Fig. S3. USP10 expression is not regulated by the HDAC7/β-catenin/c-Myc pathway. [file 40779_2022_412_MOESM1_ESM.pdf]

Table S1 Detailed information of the primary antibodies

Antibody	Brand	Cat No.
HDAC1 (10E2)	CST	#5356
HDAC2 (3F3)	CST	#5113
HDAC3 (7G6C5)	CST	#3949
HDAC4	Proteintec	17449-1-AP
HDAC5	Proteintec	16166-1-AP
HDAC6	Proteintec	12834-1-AP
HDAC7 (D4E1L)	CST	#33418
HDAC7	Proteintec	26207-1-AP
Phospho-HDAC4 (Ser246)/HDAC5 (S259)/HDAC7 (S155) (D27B5)	CST	#3443
HDAC8	Elabscience	E-AB-14127
HDAC9	Proteintec	67364-1-Ig
HDAC10	Proteintec	24913-1-AP
c-Myc (D84C12)	CST	#5605
p21	Proteintec	10355-1-AP
p27	Proteintec	25614-1-AP
Cyclin A2	Proteintec	66391-1-Ig
Cyclin B1	Proteintec	55004-1-AP
Cyclin D1	Proteintec	60186-1-AP
Cyclin E1	CST	#4129
CDK1	Proteintec	19532-1-AP
CDK2	Proteintec	10122-1-AP
CDK4	Proteintec	11026-1-AP
CDK6	Proteintec	14052-1-AP
Flag tag	Proteintec	66008-3-Ig
HA tag	Proteintec	51064-2-AP
GFP-tag	Proteintec	66002-1-IG
GST-tag	CST	#2624
β-catenin	Proteintec	66379-1-Ig
β-catenin (D10A8)	CST	#8480
Acetyl-β-catenin (Lys49) (D7C2)	CST	#9030
Phospho-β-catenin (Ser675) (D2F1)	CST	#4176
TCF4	Proteintec	22337-1-AP
USP10 (D7A5)	CST	#8501
USP10	Bioss	bs-9267R
14-3-3	Proteintec	66061-1-IG
Ubiquitin	Proteintec	10201-2-AP
LaminB1	Bioworld	BS3574
GAPDH	CST	#5174
β-actin	CST	#3700
Vinculin	Proteintec	66305-1-Ig
Flag agarose gel	Sigma	A-2220
GST beads	GE Healthcare	17-0756-05
Ni-NTA Agarose	QIAGEN	30210

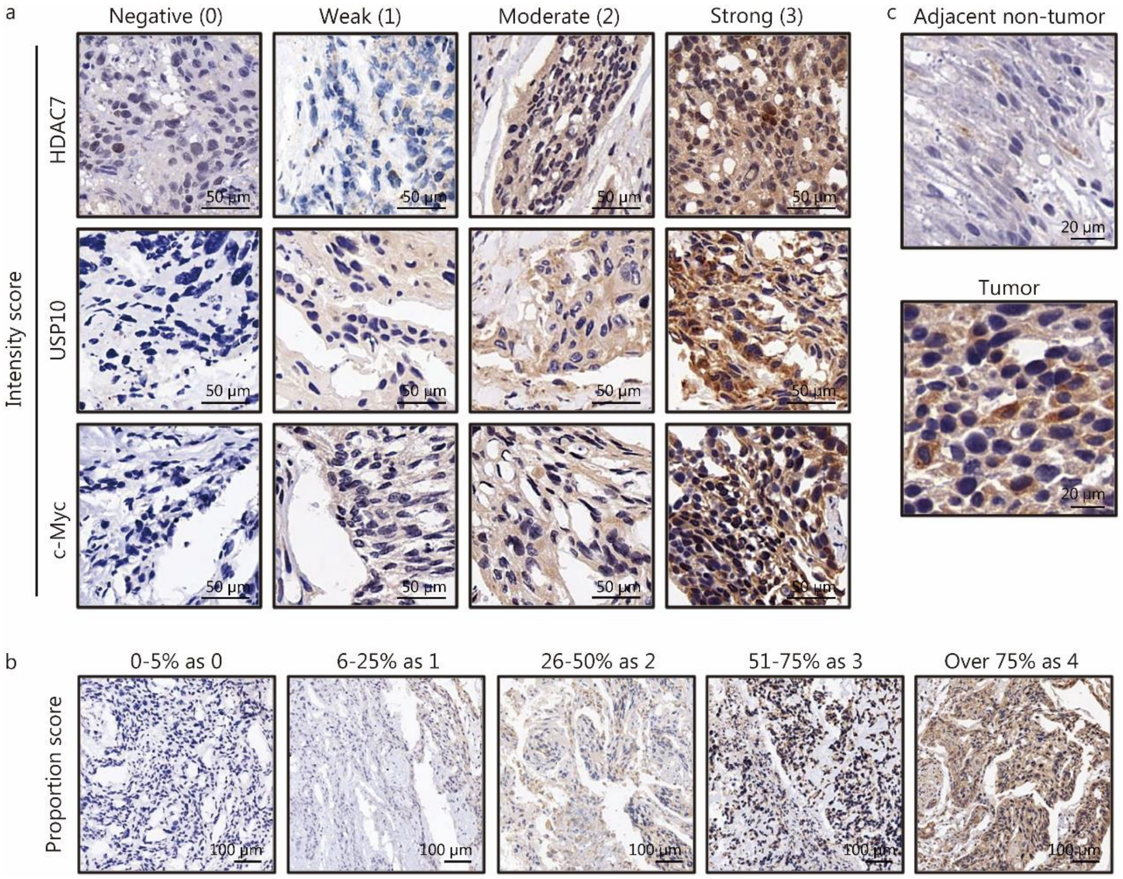

Fig. S1 Criteria of the IHC staining scores. **a** and **b** Representative HDAC7, USP10 and c-Myc IHC staining images with different intensity score and proportion score in ESCC tissues. Scale bar, 50 μm and 100 μm, respectively. **c** Representative IHC images of the ESCC adjacent non-tumor tissues and tumor tissues. Scale bar, 20 μm. HDAC7 histone deacetylase 7, IHC immunohistochemistry, USP10 ubiquitin-specific peptidase 10

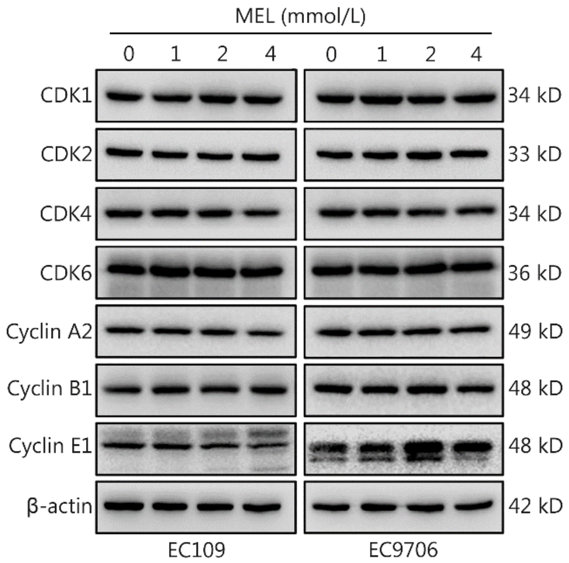

Fig. S2 Melatonin treatment for 48 h did not affect the expression of the listed cell cycle-related proteins in the EC109 and EC9706 cells. Representative Western blotting results of CDK1, CDK2, CDK4, CDK6, Cyclin A2, Cyclin B1 and Cyclin E1 were shown. CDK cyclin-dependent kinase

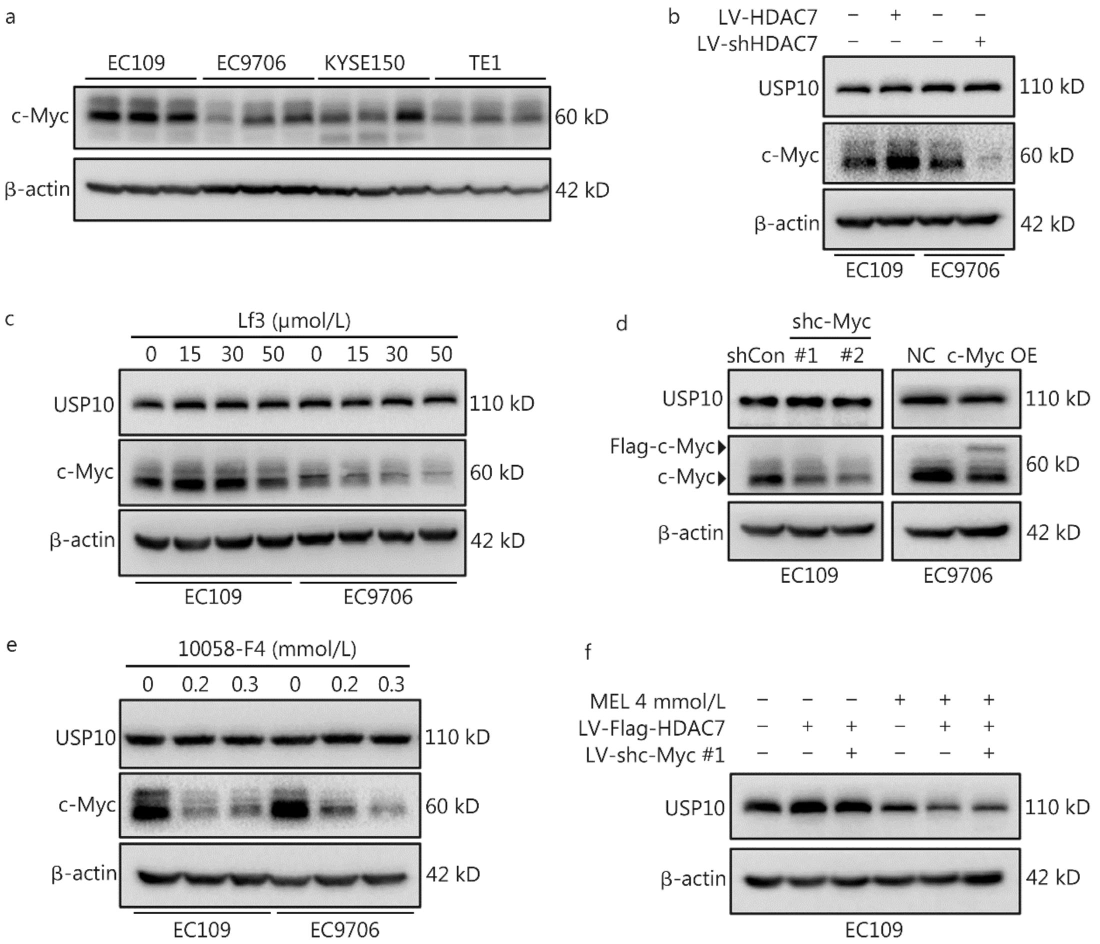

Fig. S3 USP10 expression is not regulated by the HDAC7/β-catenin/c-Myc pathway. **a** Representative Western blotting result of c-Myc expression in the different ESCC cells lines. **b** and **c** HDAC7 overexpression, knockdown or Lf3 treatment for 48 h did not affect the USP10 protein level in the ESCC cells. **d** and **e** c-Myc overexpression, knockdown or 10058-F4 treatment for 48 h did not affect the USP10 protein level in the ESCC cells. **f** Co-treatment of melatonin, LV-Flag-HDAC7 and LV-shc-Myc #1 was applied to EC109 cells. Representative Western blotting results of USP10 were shown. HDAC7 histone deacetylase 7, USP10 ubiquitin-specific peptidase 10, LV lentivirus
